# First assessment of hoary fox (*Lycalopex vetulus*) seasonal ovarian cyclicity by non-invasive hormonal monitoring technique

**DOI:** 10.1093/conphys/coaa039

**Published:** 2020-05-08

**Authors:** Ísis Zanini das Candeias, Caio Filipe da Motta Lima, Frederico Gemesio Lemos, Katherinne Maria Spercoski, Claudio Alvarenga de Oliveira, Nucharin Songsasen, Marcelo Alcindo de Barros Vaz Guimarães

**Affiliations:** Programa de Conservação Mamíferos do Cerrado, Goiás 75704 020, Brasil; Departamento de Reprodução Animal, Faculdade de Medicina Veterinária e Zootecnia, Universidade de São Paulo, São Paulo 05508 270, Brasil; Programa de Conservação Mamíferos do Cerrado, Goiás 75704 020, Brasil; Fundação Parque Zoológico de São Paulo, São Paulo 04301 002, Brasil; Programa de Conservação Mamíferos do Cerrado, Goiás 75704 020, Brasil; Departamento de Ciências Biológicas, Unidade Acadêmica Especial de Biotecnologia, Universidade Federal de Goiás / Regional Catalão, Goiás 75704 020, Brasil; Departamento de Biociências, Setor Palotina, Universidade Federal do Paraná, Paraná 85950 000, Brasil; Departamento de Reprodução Animal, Faculdade de Medicina Veterinária e Zootecnia, Universidade de São Paulo, São Paulo 05508 270, Brasil; Center for Species Survival, Smithsonian Conservation Biology Institute, Front Royal, VA 22630, USA; Departamento de Reprodução Animal, Faculdade de Medicina Veterinária e Zootecnia, Universidade de São Paulo, São Paulo 05508 270, Brasil

**Keywords:** faecal metabolites, Neotropical canids, oestrogen, progestagen, reproduction, reproductive hormones

## Abstract

Reproduction is key to species survival, and reproductive physiology represents a high priority investigative area for conservation biology, as it provides a basic understanding of critical life-history traits, information that is helpful for the establishment of management strategies. Here, we generated knowledge about the reproductive endocrinology of the hoary fox (*Lycalopex vetulus*), a small canid (2.5–4 kg) endemic to open areas of the Brazilian Cerrado and listed in the Brazilian National List of Endangered species. Specifically, we utilized non-invasive hormone monitoring methods to assess oestrogen and progestagen metabolites from eight female hoary foxes housed in five zoological institutions in the state of São Paulo—Brazil. We observed the elevations of oestrogen and progestagen metabolites between July and September in six of the eight females. No significant evidence of ovarian activity was observed during other months. Two females, who shared the same enclosure, did not show a pattern of reproductive cyclicity. Based on these characteristics, we concluded that captive hoary foxes are seasonal monoestric, with the beginning of the oestrus cycle occurring mainly in July followed by 2 months of the luteal phase when conception does not occur. We suggest the dosage of faecal metabolites of estradiol and progesterone could be used to differentiate the reproductive period from a non-reproductive period in *Lycalopex vetulus* females, providing relevant information about their reproductive biology that may contribute to species conservation and management strategies, such as increased *ex situ* reproductive success.

## Introduction

Reproduction is key to species survival, and reproductive physiology represents a high priority investigative area for conservation biology, as it provides a basic understanding of critical life-history traits, information that is helpful for the establishment of management strategies. (Wildt and Wemmer, 1999; Bush and Hayward, 2009; Comizzoli *et al*., 2009).

It is well established that there are significant variations in reproductive mechanisms among species across taxa (Brown, 2018). As a result, it is essential to utilize a species-specific approach for developing effective reproductive monitoring, management and assisted reproduction techniques (Wildt and Wemmer, 1999; Brown, 2018). Such tools are keys to the long-term sustainability and genetic viability of *ex situ* and *in situ* wildlife populations (Conde *et al.*, 2013).

The Iberian lynx (*Lynx pardinus*) is an example of how the knowledge on reproductive physiology can help in structuring integrated conservation programs. Until 2005, this species could not reproduce in captivity, presenting high incidences of abortion and premature birth, and a non-invasive pregnancy test was required. In lynx, however, faecal steroid profiles do not follow the typical pregnancy pattern of felids. The discovery that urine and faecal samples of pregnant females contain significantly elevated levels of PGFM, a metabolite of prostaglandin-F2α, 3 weeks prior to parturition was the breakthrough for establishing a reliable and straightforward pregnancy diagnosis, that posterior was used for several feline species. This diagnosis and the knowledge on the physiological particularities of this species allowed the use of assisted reproduction techniques, leading to an increase in captive breeding success and contributing to downgrading its threat status from ‘Critically Endangered’ to ‘Endangered’ accordingly to IUCN (International Union for the Conservation of Nature) (Jewgenow *et al*., 2017).

Here, we aim to characterize the reproductive physiology of the hoary fox (*Lycalopex vetulus*), a small canid (2.5–4 kg) endemic to open areas of the Brazilian Savannah biome, the Cerrado (Dalponte and Courtenay, 2004; Dalponte, 2009; Lemos *et al*., 2013). Although assessed by the IUCN as ‘Least Concern’ (Dalponte and Courtenay, 2008), the Brazilian Red List Assessment highlights the species as *vulnerable to extinction* (Lemos *et al*., 2013). The main threats for this small canid are anthropogenic actions, including road killing, poisoning, retaliatory hunting and persecution by domestic dogs. Conversions of natural habitats in long-scale agriculture also pose a threat to the species (Lemos *et al*., 2011b; Lemos *et al*., 2013; Lemos, 2016).

Hoary foxes live in monogamous pairs during the whole year and in family groups during pup rearing and part of the predispersion period (Courtenay *et al*., 2006; Dalponte, 2009; Lemos, 2016). The species forage solitarily for most of the year but maybe found foraging in pairs or with pups (Lemos and Facure, 2011). The diet is a typical omnivorous with consists predominantly of termites, followed by other arthropods, fruits and small vertebrates (Courtenay *et al*., 2006; Dalponte, 2009; Kotwiski *et al*., 2019).

Despite some studies on its diet, behaviour and home range (Juarez and Marinho-Filho, 2002; Dalponte, 2003; Courtenay *et al*., 2006; Lemos, 2007; Lemos and Facure, 2011; Lemos, 2016), little is known about the species’ physiology, and to our knowledge, there are no studies on the reproductive physiology of any species of the *Lycalopex* group. However, from wild observations, we know that the reproductive period of this species is markedly seasonal, with births occurring once a year from late July to September (Courtenay *et al*., 2006; Dalponte, 2009; Lemos *et al*., 2013, Lemos, 2016). The precise duration of gestation is unknown but mating in the wild occurs between May and July gave a gestation around 50 days, with two to five pups per litter (Courtenay *et al*., 2006; Dalponte, 2009). Pups are born in a den and cared for by both parents (Courtenay *et al*., 2006; Dalponte 2009; Lemos *et al*., 2013; Lemos, 2016).

This work intends to generate primary data about the reproductive endocrinology of the hoary fox, to better understanding and compare the reproductive biology of this threatened canid. The objectives of the present study were to (i) validate non-invasive hormonal monitoring techniques (extraction and measurement of faecal progestagen (FPM) and oestrogens (FEM) metabolites by enzyme immunoassay—EIA) for hoary foxes; (ii) characterize gonadal steroids patterns during the ovarian cycle in captive individuals; and (iii) evaluate differences between the concentrations of these metabolites in a period of 12 months.

## Materials and methods

### Animals and sample collection

Eight females housed in five zoological institutions were included in the study. The age of animals was based on information provided by the institutions and ranged from two to 12 years. Only two females (F4 and F5) had a history of breeding in captivity (Table 1). None of the institutions use contraception methods for hoary foxes, mainly because of their difficulty in breeding the species. Of the eight females, one was housed with a male; however, pregnancy and breeding were not observed during the study. All animals underwent health examination by a veterinarian at each institution at the beginning of the study and appeared to be healthy and well-nourished.

**Figure 1:**
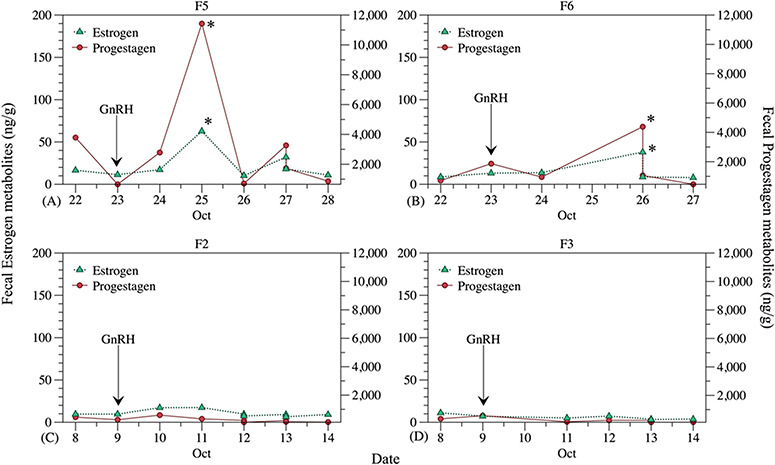
Physiological validation for EIA assays for oestrogen and progesterone metabolites from four captive female hoary foxes (*Lycalopex vetulus*). Arrows indicate the application of GnRH, and asterisks (*) the peaks of faecal metabolites

**Table 1:** List of captive hoary foxes (*Lycalopex vetulus*) assessed to characterize the ovarian cyclicity

**Female ID**	**Age (year)**	**Institutions**	**Latitude/longitude**	**Enclosure size (m** ^**2**^ **)**	**Diet**	**Feeding frequency**	**Housing status**	**Reproducing in captivity**
F1	>7	Americana Ecological Park –PEA	22° 45′ 7.98″ S/47° 21′ 10.49″ W	61.5	Dog food, beef, beef guts, apple, banana, papaya, carrot and beet Water ad libitum	Once a day in the morning	With a male	No
F2	>8	Piracicaba Zoo—ZP	22° 41′ 41.98″ S/47° 39′ 5.52″ W	70	Dog food, beef, banana, papaya, carrot, beetroot, egg, pineapple and avocado Water ad libitum	Fruits and vegetables in the morning, meat in the afternoon	With a female	No
F3	>3	Piracicaba Zoo—ZP	22° 41′ 41.98″ S/47° 39′ 5.52″ W	70	Dog food, beef, banana, papaya, carrot, beetroot, egg, pineapple and avocado Water ad libitum	Fruits and vegetables in the morning, meat in the afternoon	With a female	No
F4	12	Ecological Park Dr Antônio Teixeira Vianna—PESC	21° 59′ 5.29″ S/47° 52′ 32.34″ W	200	Dog food, beef and chicken, banana, papaya, mouse, termites and sugar cane Water ad libitum	Once a day in the morning	Alone	Yes
F5	>5	Quinzinho de Barros Municipal Zoo—PZMQB	23° 30′ 19.31″ S/47° 26′ 19.67″ W	68	Dog food, beef guts, banana, papaya, pineapple, watermelon, mouse and chick Water ad libitum	Once a day in the morning	With a female	Yes
F6	>5	Quinzinho de Barros Municipal Zoo—PZMQB	23° 30′ 19.31″ S/47° 26′ 19.67″ W	68	Dog food, beef guts, banana, papaya, pineapple, watermelon, mouse and chick Water ad libitum	Once a day in the morning	With a female	No
F7	3	Municipal Zoo Dr Fábio de Sá Barreto—ZMFSB	21° 10′ 22.41″ S/47° 48′ 10.24″ W	10	Dog food, beef, banana and papaya Water ad libitum	Once a day in the morning	With a female	No
F8	2	Municipal Zoo Dr Fábio de Sá Barreto—ZMFSB	21° 10′ 22.41″ S/47° 48′ 10.24″ W	10	Dog food, beef, banana and papaya Water ad libitum	Once a day in the morning	With a female	No

Legend: (>) represents animals whose age historic is unknown by institution; the number represents estimated age

Enclosure sizes ranged from 10 to 200 m^2^, with the smallest (housing F7 and F8), the only non-exhibition enclosure, being located in an animal receiving area. All animals were fed at least once a day. The diet generally consisted of commercial dog food, beef, chicken viscera, fruits and vegetables (Table 1). Access to water was *ad libitum* for all animals.

Fresh faecal samples were collected in the morning, between 8 a.m. and 10 a.m., depending on the management of each zoo, three times a week for 12 months. Animals that shared the same enclosure were kept separate at night (defecation period) for the identification of faeces. Samples were labelled and kept at −20°C freezer until hormone extraction in the Laboratory of Hormonal Dosages at the Veterinary School—São Paulo University, Brazil (LDH-FMVZ/USP).

This research was conducted under the approval of the Ethical Committee for the Use of Animals of the University of São Paulo (no. 0.2714/2002).

### Extraction of metabolites

Faeces extractions were performed according to the recommended by Palme and Möstl (1996) adapted by LDH-FMVZ/USP, as follows: 0.2 g of lyophilized faeces was weighed and transferred to a properly identified test tube; 5 ml methanol was added with 80% concentration (80% methanol and 20% ultrapure water); tubes were placed in a multi-vortex apparatus (VWR Scientific Products, VX-2500) for 15 min; centrifugation was done for 20 min at 2200 rpm (Universal 320—Hettich Zentrifugen); and 1.0 ml of the supernatant (faecal extract) was transferred to a 2.0-ml conical plastic tube and then dried in air. Dried tubes were sealed and stored in a −20°C freezer. Extracted samples were transported to the Smithsonian Conservation Biology Institute (SCBI), Virginia, USA (MAPA certificate no. 005/2014/SUS GR) for hormonal analysis.

### EIA protocol

Dried samples were re-suspended in 1.0 ml of the dilution buffer solution (NaH_2_PO_4_—0.04 M; Na_2_HPO_4_—0.06 M; NaCl—0.15 M; pH: 7.00). Assays utilized a monoclonal progesterone antibody (Quidel CL425, C.J. Munro, University of California, Davis, CA) and polyclonal antibodies to E1 polyclonal glucuronide R522-2b (C.J. Munro, University of California, Davis, CA) in the oestrone conjugate assays. The dilutions used were 1: 50 000 and 1: 40 000 for antibodies CL425 and R522-2b, respectively. The respective dilution of HRPs (Sigma-Aldrich, St. Louis, MO) was 1: 75 000 and 1: 80 000, respectively. Plates (Nunc-Immuno, Thermo Fisher) were kept at room temperature ~30 min before use 50 μl of standard solution or control per well in duplicate. After 25 μl/well of HRP and 25 μl/well of antibody were added, microplates were sealed and incubated for 2 h at room temperature. After the incubation, microplates were washed five times with wash solution (Sigma-Aldrich), dried, and 100 μl/well of a chromogenic substrate (TMB substrate) was added. The chromogenic reaction was stopped with 100 μl/well STOP solution (H_2_SO_4_ 96%: 10%) after 30 min for progesterone assays and 6 min for estradiol assays. Dynex MRX, Dynex Technologies—VA, was used to read plates using a 450-nm filter. All samples, controls and standards were performed in duplicates.

Intra-assay coefficients of variation (CVs) (*n* = 1091), made individually for each sample, were below 10% (min 0.00 and max 9.11). The inter-assay CV (*n* = 127), using the mean values of duplicates of the control samples (high control around 30% binding; low control around 70% binding) were <12%. Inter-assay CV for progestagen assays was 7.56 and 11.34% (high and low controls, respectively), and that for estradiol assays was 6.32 and 8.35% (high and low controls, respectively). The solutions used to make the high and low controls were the known standards, used in dilutions to achieve 30 and 70% binding. Hormone data are reported as μg/g faeces. For each assay, the parallelism and recovery tests were performed.

### Technical validation of enzyme immunoassay

Technical validation of the assays was also performed. Briefly, the steroid hormone metabolites were extracted from dried faecal samples (10 samples randomly selected), and aliquots from the extract were pooled.

Serial dilutions of pooled faecal extracts produced displacement curves significantly parallel to those of the appropriate standards. The comparison was made through linear regression between the percent binding of the extracts and standards (*P* < 0.05, progesterone *R*^2^ = 0.994, estrone conjugate *R*^2^ = 0.988). Recovery for each EIA was determined by addition of known concentrations of standard to the pools and comparison through regression of the mass added to the pool versus the mass measured in each assay. Recovery of added standard to pooled faecal extracts demonstrated significant (*P* < 0.05) recovery (progesterone, *y* = 0.81*x* − 7.23, *R*^2^ = 0.997; and estrone conjugate, *y* = 0.65*x* − 0.44, *R*^2^ = 0.988).

Also, high-performance liquid chromatography (HPLC) was used to identify immune reactive metabolites in a pooled sample relative to the radioactively labelled hormone (see below).

### Physiological validation

The physiological validation of the assays for the determination of faecal metabolites of steroids was performed using Females 2, 3, 5 and 6. Fresh faeces were collected for seven consecutive days, between 8 a.m. and 10 a.m., depending on the management of each zoo, in October (non-reproductive season). After the second day of faeces collection, a single injection of 0.0084 mg of buserelin acetate, a synthetic analogue of GnRH (Sincroforte®—Ourofino; 0.0024 mg/kg body weight), was administered intramuscularly in the morning (8 a.m.).

### HPLC protocol

HPLC analysis (Varian ProStar; Varian Analytical Instruments, Lexington, MA) was performed to determine numbers and proportions of immunoactive steroid hormone metabolites present in hoary fox faeces using previously published protocols (Brown, 2008).

Resulting pooled extracts of 10 samples selected randomly from different animals were re-suspended in 0.5 ml PBS (Na_2_HPO_4_—0.03 M, NaH_2_PO_4_–0.02 M, NaCl—0.15 M, NaN_3_—0.002 M, pH: 5.0), filtered through a C18 Spice cartridge (#01–10, Analtech Inc., Newark, DE), and dried under forced air. Radioactive tracers (300 μl; tri-estrone sulphate, estradiol, estrone, progesterone) were added to the appropriate pooled sample as chromatographic markers and dried again. The extract was reconstituted in 0.3 ml methanol and sonicated for 5 min, and 0.05 ml was loaded onto a reversed-phase C18 HPLC column (Agilent Technologies, Santa Clara, CA). For progestagen, the sample was separated using a 20–100% linear gradient of acetonitrile: water over 120 min (1 ml/min flow rate; 1-ml fractions). For oestrogens, samples were separated using a 20–80% linear gradient of methanol: water over 80 min (1 ml/min flow rate; 1-ml fractions). An aliquot of each fraction was counted on a multi-purpose β-radiation scintillation counter (LS 6500, Beckman Coulter, Brea, CA), and the remainder of each fraction was performed EIA analysis. Each fraction was analyzed in single, and retention times of radioactive markers and immunologic activity were compared to identify hormone metabolites.

### Statistical analysis

Baseline oestrogen metabolite values were calculated for each individual by an iterative process, whereby high values (exceeding the mean plus 1.5 SD) were excluded. Each time the average was recalculated, and the elimination process repeated until no values exceeded the mean plus 1.5 SD, those remaining were considered baseline values (Brown *et al*., 1996; Wielebnowski *et al*., 2002; Khonmee *et al*., 2017). The same iterative process, excluding values that exceeded the mean plus 2.0 SD, calculated baseline progesterone metabolite values. The use of 2.0 SD in the progesterone analysis resulted in a more biologically relevant estimate of baseline values compared to 1.5 SD (Moreira *et al*., 2001; Luaces *et al*., 2011). This process of successive exclusion was used only to establish the baseline values; no data were excluded from statistical analysis and descriptive results. The highest value within a cluster of three consecutive values above the baseline was considered a peak. Values are presented as mean ± SEM.

Data were tested for normality of residues (normal distribution) and homogeneity of variances. Faecal oestrogen metabolites values had non-Gaussian distribution and were normalized applying a logarithm (base 10). Differences in faecal metabolites concentration of cycling females among different months were determined using Kruskal–Wallis one-way analysis of variance (ANOVA) followed by least significant difference (LSD) test for multiple comparisons. Differences were considered significant when the *P* value was < 0.05. Data were analyzed using the SAS System for Windows 9.3 (SAS, 2000).

To analyze the data based on the reproductive phases, the longitudinal steroid metabolite profiles were aligned based on the day of the FEM peak that was considered day zero (Day 0). In females that lacked a distinctive FEM peak, Day 0 was identified as the first day that FEM rises above basal concentrations. Because faecal samples were not always available, the data across an individual’s profile were grouped into 3-d means.

Longitudinal profiles of each faecal steroid metabolites were then divided into three reproductive phases, using the criteria described by Songsasen *et al.* (2006): (i) basal phase (from Day −30 to Day −10; (ii) periovulatory phase (from Day −9 to Day +9); and (iii) luteal phase (from Day +10 to Day +75). Differences in faecal steroid metabolite concentrations between each phase were determined using the Kruskal–Wallis one-way analysis of variance (ANOVA) followed by Dunn’s multiple comparison test. Differences were considered significant when the *P* value was < 0.05. Data for this analysis was made using GraphPad Prism 8.3.0.

## Results

During 12 months, a total of 1091 faecal samples were collected from eight captive female hoary foxes with an average of 136.5 (± 11.04 SEM) samples per individual and 91 (± 4.74 SEM) samples per month. None of the females became pregnant during this period.

### Physiological validation

Physiological validation was successful for two (F5 and F6) of four females. Peaks in faecal progestagen and estradiol metabolites were detected between 48 and 72 h after the GnRH application. Females that did not reach the peak (F2 and F3) probably were no longer in the follicular phase, but at the beginning of the anestrus, not enabling the stimulation. Nevertheless, results from F5 and F6 are substantial to demonstrate that assays used were able to detect changes in the levels of faecal steroid metabolites compared to respective changes of steroid concentrations in the blood (Fig. 1).

### HPLC

**Figure 2:**
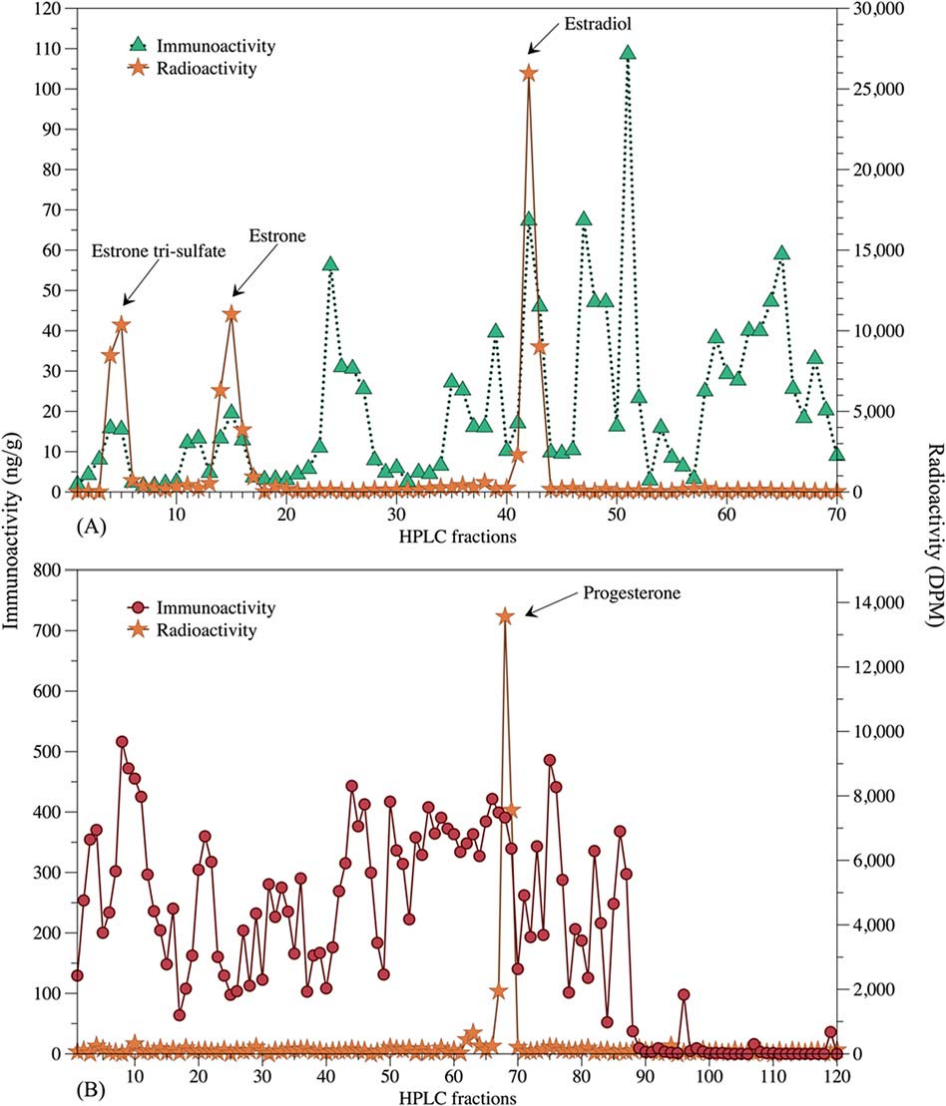
HPLC profiles and enzyme-immunoassay dosage of fractions generated on HPLC and radioactive counting of the same fractions for oestrogen (**A**) and progesterone (**B**) metabolites in pooled faeces from eight captive female hoary foxes.

Evaluation of faecal extracts by HPLC revealed the presence of several metabolites. HPLC analysis of oestrogen metabolites demonstrated three immune-reactive peaks (Fractions 4 and 5, 13 to 17 and 41–43), which co-eluted with estrone-3-sulphate, estrone and estradiol (Fig. 2A). Progestagen immune-reactivity was associated with a single peak (Fractions 67 to 69), which was corresponded to the radio-labelled progesterone (Fig. 2B).

### Faecal metabolites of oestradiol and progesterone dosage

Mean baseline for FEM and FPM were 12.07 ng/g (± 0.44 SEM) and 762.44 ng/g (± 146.94 SEM), respectively, and mean peak values for FEM and FPM were respectively 37.78 ng/g (± 2.62 SEM) and 6026 ng/g (± 1415 SEM). The individual baseline and peak mean are shown in Table 2.

**Table 2:** Baseline and peak mean concentrations of faecal oestrogens and progestagen metabolites for individual hoary foxes in samples collected for 12 months

Female ID	Faecal oestrogen metabolites		Faecal progestagen metabolites	
	Baseline (ng/g)	Peak mean (ng/g) ± SEM	Baseline (ng/g)	Peak mean (ng/g) ± SEM
F1	10.97	37.78 ± 3.79	695.65	8244.81 ± 989.71
F2	14.05	44.05 ± 9.19	422.93	4673.52 ± 861.03
F3	12.72	37.18 ± 4.56	350.65	3192.18 ± 449.83
F4	10.82	36.02 ± 6.04	447.21	3551.90 ± 472.49
F5	11.71	40.89 ± 7.92	1176.43	4417.31 ± 296.98
F6	13.64	31.61 ± 3.38	1451.88	12075.51 ± 1555.7
F7	11.50	NA	1071.80	NA
F8	11.18	NA	482.95	NA

NA, not applicable. The mean peak for both hormones was calculated only for cycling females.

**Figure 3:**
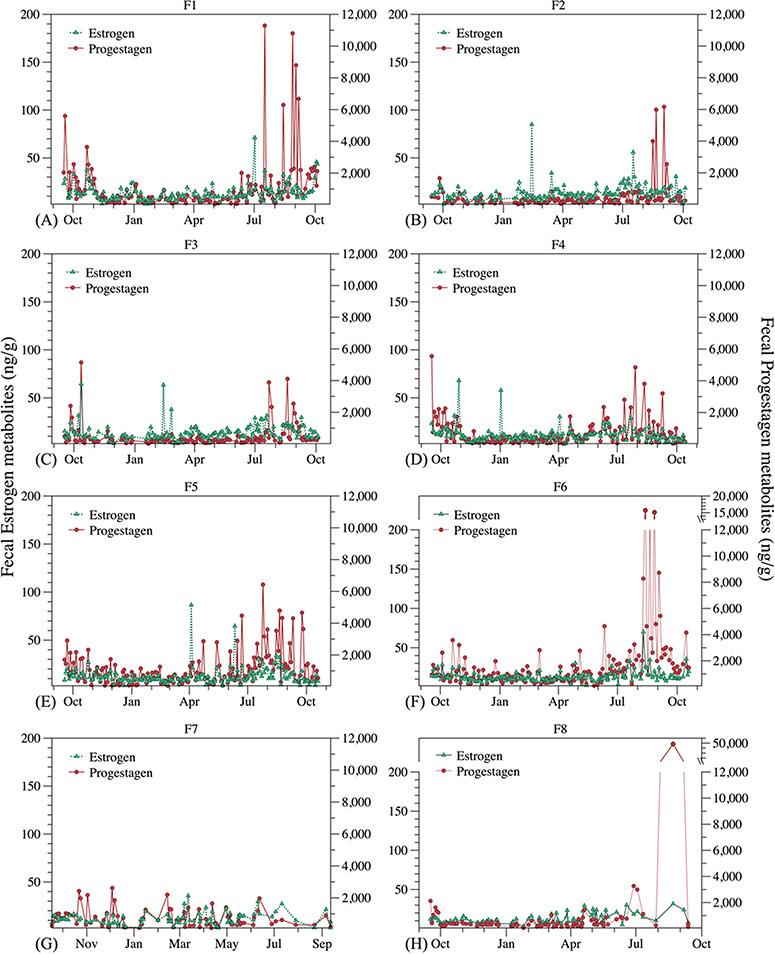
Longitudinal profiles of faecal oestrogen and progestagen metabolites from non-pregnant captive hoary foxes (F1–F8) along 12 months, between September 2012 and September 2013.

Six out of eight female hoary foxes displayed periods of elevated oestrogen (July and August) and progestagen faecal metabolites secretion (July–September). Females 1, 3, 4 and 5 had elevated oestrogen followed by elevated progestogen in July, and Females 2 and 6 exhibited this hormone pattern in August. Two individuals (F7 and F8), from the same zoo, did not show any sign of ovarian cyclicity (Fig. 3). There was no difference in the excretion profile of faecal metabolites of oestrogen and progestagen in relation to the presence of conspecifics in captivity (single females, female with other female or female with a male).

**Figure 4:**
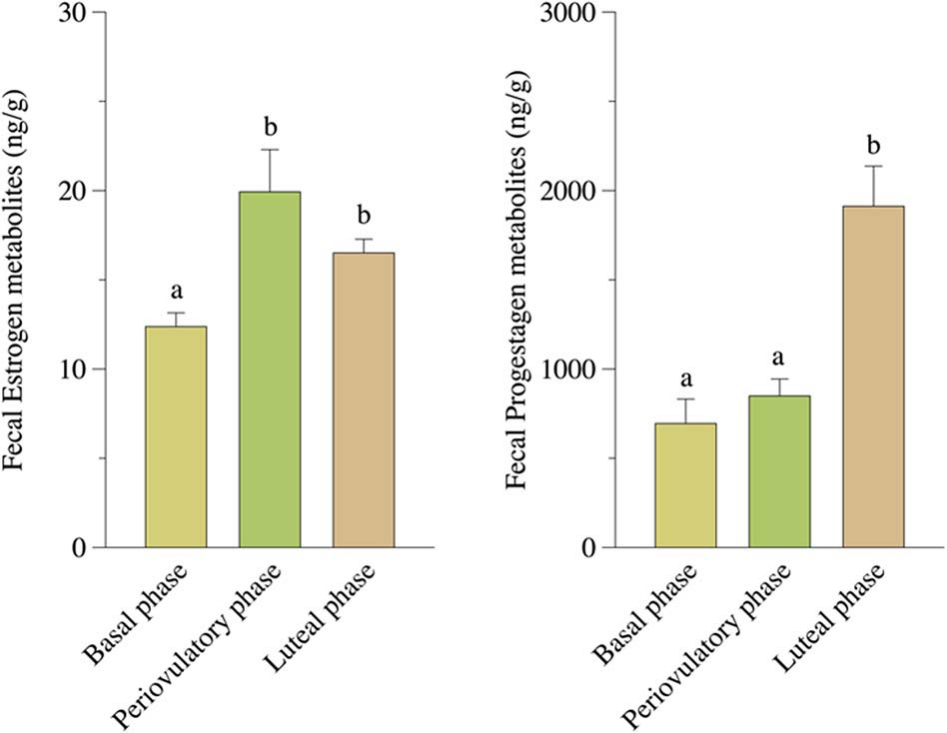
FEM and FPM concentrations across reproductive phases in cycling female hoary foxes. Different letters show a statistical difference from the groups (*P* < 0.05). Vertical bars indicate SEM

**Figure 5:**
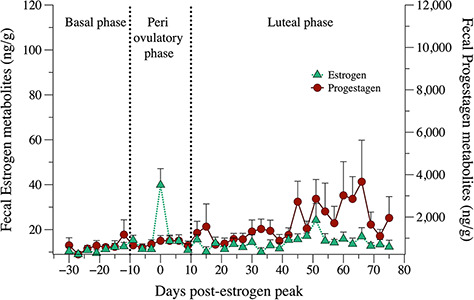
Three-day mean (± SEM) longitudinal profiles of FEM and FPM in cycling female hoary foxes

The result of the reproductive phases is shown in Fig. 4. In cycling females, FEM concentrations were lower during basal phase (12.38 ng/g ± 0.78 SEM; *n* = 39) and rise from periovulatory phase (19.93 ng/g ± 2.37 SEM; *n* = 38) to luteal phase (16.51 ng/g ± 0.76 SEM; *n* = 117). For FPM, the basal phase (694.60 ng/g ± 137.00 SEM; *n* = 39) and the periovulatory phase (849.10 ng/g ± 94.74 SEM; *n* = 38) showed no differences, but the levels rise to the luteal phase (1912.00 ng/g ± 224.70 SEM; *n* = 117). The longitudinal profiles for FPM and FEM of cycling females are shown in Fig. 5.

**Figure 6:**
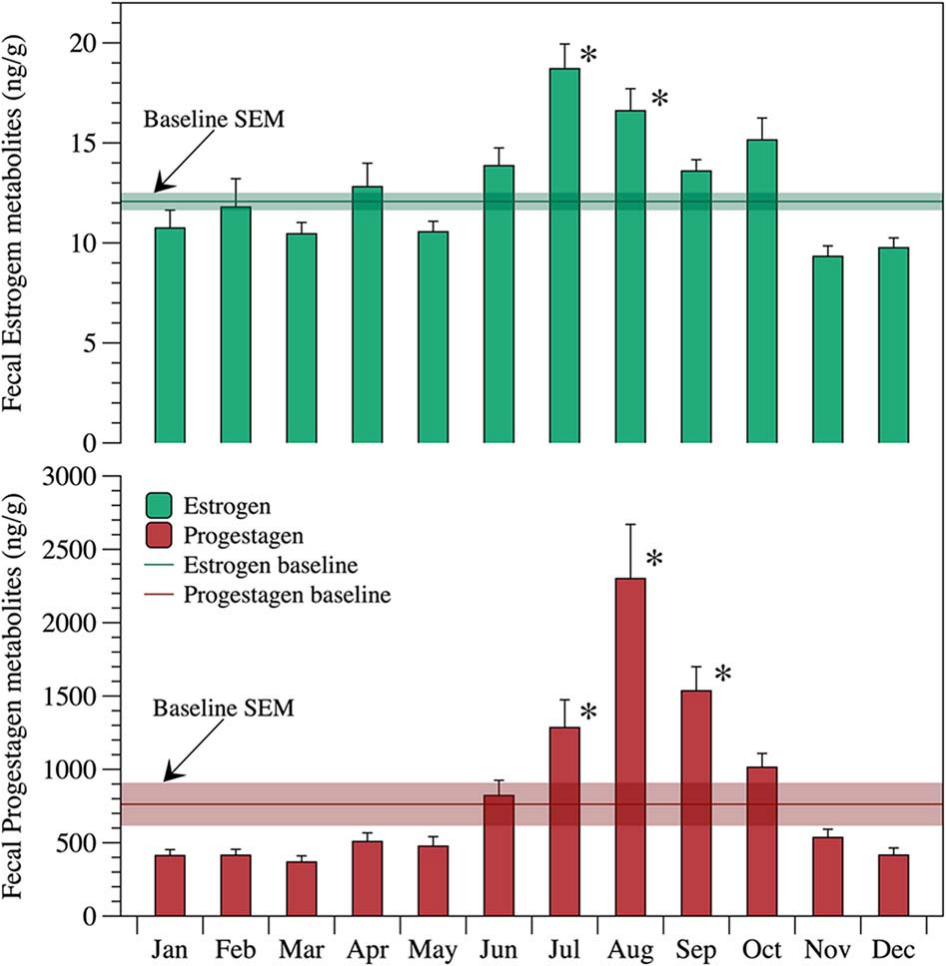
Monthly means of faecal oestrogen and progestagen metabolites for six captive cycling female hoary foxes. The asterisk symbol represents months with the highest average and statistical difference from other months for each hormone. (*P* < 0.0001). Vertical bars indicate SEM

In cycling females, both oestrogen and progestagen metabolites concentrations gradually increased starting June, reached their higher levels for oestrogen during July and August, and between August and September for progestagen, and then both declined to baseline from November to May (Fig. 6).

## Discussion

To date, there is no information about the reproductive endocrinology of the hoary fox. In the present study, we utilized non-invasive hormonal monitoring to longitudinally assess faecal oestrogen, and progestagen metabolites in female hoary foxes maintained in Brazilian zoos. Our data indicate that captive female hoary foxes are monoestrous with a seasonal oestrus cycle, with the beginning of oestrus occurring mainly in July followed by a 2-month period (August and September) of the luteal phase when conception does not occur.

The hoary fox ovarian cyclicity presented hormonal events similar to that reported for most canid species. The *Canidae* reproductive biology includes many unusual features, some even unique, from other mammals (Kleiman, 1977; Asa and Valdespino, 1998). Most canids studied to date share similar particularities in their reproductive physiology, including (i) seasonal ovarian activity and for most species only once a year. The exception for this is the Asian wild dog (*Cuon alpinus*), described to have a seasonal reproductive cycle but indicate to be a polyestrous canid, characteristic that differs it from others within the Canidae family (Khonmee *et al*., 2017); (ii) preovulatory rise in serum progesterone (Verstegen-Onclin and Verstegen, 2008; Concannon, 2009; Concannon, 2012); and (iii) obligatory period of anestrus (Asa and Valdespino, 1998; Concannon, 2009). The domestic dog (*Canis lupus familiaris*) is the canid with the greatest amount of information about its reproductive cycle, and knowledge from this species can be applied and compared to wild species. Therefore, regarding fluctuations of oestrogens and progestagens, hoary foxes presented hormonal events and reproductive cycle similar to that reported for most canids, including domestic dogs.

The periovulatory phase in assessed hoary foxes was marked by increased concentrations and frequency of FEM peaks. Later, during the luteal phase, FPM concentrations rise and remain elevated, followed by basal phase, which we could consider an anestrus period. Hormone profiles observed on female hoary fox were very similar to hormonal events that occur in domestic dog reproductive cycle, with an elevation of circulating oestrogens rising from the late anestrus to the late proestrus, and serum progesterone concentration remains low until the end of proestrus when the hormone level begins to rise (Verstegen-Onclin and Verstegen, 2008; Concannon, 2009; Concannon, 2012).

Similar reproductive profiles were also described to two other Brazilian canids, sympatric to the hoary fox, both the crab-eating fox (*Cerdocyon thous*) and the maned wolf (*Chrysocyon brachyurus*) present seasonal monoestric cycle between autumn and winter. The crab-eating fox has a similar reproductive season that goes approximately from June to September. After a gestation period of 58–60 (± 3) days, pups are born from August on (Souza *et al*., 2012). Captive maned wolves have a well-known distinctive 3–5-month breeding season that occurs from April to June in Latin America and October to February in North America (Velloso *et al*., 1998; Songsasen *et al*., 2006). Proestrus rangees from 3to 18 days, with estrous duration ranging from 1 to 10 days, and gestation period of ~65 days. As registered for captive hoary foxes, maned wolf progestagens remained baseline until proestrus, when a steep increase in progestogen levels was observed in the profiles of cycling females, and the hormone concentration remained elevated throughout pregnancy and pseudopregnancy (Velloso *et al*., 1998; Songsasen *et al*., 2006).

Two females (7 and 8), which shared the same enclosure at Ribeirão Preto Zoo, did not present any cyclicity pattern. The lack of ovarian cyclicity observed in these two females could be due to several reasons. First, it could be due to chronic stress (Nelson, 2005; Dickens and Romero, 2013), since theses foxes lived in a small enclosure (10 m^2^) with no hiding places, they had visual contact with other predators (e.g. pumas, *Puma concolor*) and heavy staff traffic around them. Nevertheless, assessing faecal glucocorticoids in these females is warranted to confirm this hypothesis. Another possible reason could be the absence of male effect; both individuals arrived as pups from the wild and have never had contact with a conspecific male (Zoo personal communication). In some species, such as marmosets (*Saguinus oedipus*), for females to enter puberty and initiate their reproductive activity, the male presence is necessary (Widowski *et al*., 1992). Bush dog females (*Speothos venaticus*) do not need the male to initiate oestrus, but male presence significantly decreases interestrous interval (Dematteo *et al*., 2006). Since any additional health and reproductive exams were not performed in this study, we cannot eliminate possible health problems such as nutritional disorders, and reproductive diseases, especially those contagious or related to management.

To our knowledge, there is no previous study on the reproductive physiology of any of the six species from the *Lycalopex* groups. Furthermore, there are no reproductive hormonal parameters determined for zoo-housed hoary fox nor the other five species. Apparently, this is a difficult genus to breed in captive, with less than 15 birth records in Species 360 (ZIMS® version 2.25.5, accessed November 2019) for all six species. Fourteen of these births (93%) occurred from July to November and one (0.7%) in January (*Lycalopex sechurae*). Although sparse, these data corroborate our findings of a seasonal reproduction period occurring mainly in winter for this South American canid group.

In general, our results are close to the observed in wild populations, which have described hoary foxes as seasonal breeders, with births occurring once a year (Dalponte, 2009; Lemos *et al*., 2013). In different regions of Central Brazil, observations suggest births normally occur from August (4 l) to September (2 l) and occasionally July (1 l) (Dalponte, 2003; Courtenay *et al*., 2006; Dalponte, 2009). Although there is no precise information about the gestation period of hoary foxes, Dalponte (2003) estimated it in around 50 days. Therefore, based on estimated reported births, mating is expected to occur between June and July, and possibly May. Our results from captive foxes’ hormonal profile suggest females would be able to mate in July. Previous studies on free-ranging populations of hoary foxes (Dalponte, 2003; Courtenay *et al*., 2006; Dalponte, 2009) had not assessed physiological parameters nor observed copulation and thus making the comparisons between *in situ* and *ex situ* cycles difficult. Despite that, some aspects could explain the difference between reproductive periods. All captive females of the present study were located at São Paulo state, in different latitudes from assessed wild populations. This could lead to small variations in major ovarian activity (Bronson, 2009). Besides, living in captivity, with different food, management and activity patterns, could lead to endocrinological variations. Nevertheless, little information on the reproductive aspects of this species, either in captivity or in the wild, makes further comparisons or explanations difficult to establish. Therefore, we highlight the importance and need of more studies to gather baseline information on mating and gestation periods, behavioural endocrinology and other general aspects of the reproductive biology of wild hoary foxes and other *Lycalopex*.

The seasonality found in this study and observed in wild populations is more commonly observed in species living in temperate zones than in tropical ones, but all animals have to deal with changing season/environment to some degree (Heldstab *et al*., 2018). The hoary fox distribution ranges from latitude 4°S to 22°S; with a similar range (7°S to 25°S), wild dogs (*Lycaon pictus*) are seasonal, and this strategy has been related to assuring adequate temperature for raising pups (McNutt *et al*., 2019). For hoary foxes, environmental synchronization may be related to the higher availability of the main food resource for the species, termites. The swarming flights of termites are a seasonal phenomenon (Prestes, 2012) and benefit several predators, including hoary foxes, by the intense synchronized release of winged reproductive adults (Lemos *et al*., 2011b). Therefore, breeding in the winter could maximize their reproductive outcomes and increase the chances of offspring survival.

Canids are unique, being monoestrous and exhibiting spontaneous ovulation at the same time. This combination limits females to a single conception opportunity per year, increasing the risk of losing the breeding season if these are not able to find a partner during this limited window of reproductive activity. These characteristics raise the value of long-term pair-bonding (Macdonald *et al*., 2019). Hoary fox males have a determining role in rearing the offspring (Dalponte 2009; Courtenay *et al*. 2006; Lemos, F.G. & Azevedo, F.C. personal communication), making stable, monogamous couples even more essential to guarantee offspring success.

From a conservation perspective, being seasonal, monoestric and monogamous are potentially risky reproductive strategies in the Anthropocene changing world. Although it is the same strategy adopted with success by most canids (Macdonald *et al*., 2019), the hoary fox is endemic to the threatened Brazilian Cerrado and relies on a specialized diet (Dalponte, 2009; Kotwiski *et al*., 2019). Considered *vulnerable to extinction* by the Brazilian Red List, rapidly losing natural habitats is among the main threats to the hoary fox and forces it to inhabit agroecosystems with strong human influences (Lemos *et al*., 2011a; Lemos *et al*., 2013). Maintaining a pair through the entire reproductive season, from pairing to offspring dispersion, may be challenging in a fragmented landscape permeated by poisoning and persecution by humans, domestic dogs, and roads (Lemos *et al*., 2011a; Lemos *et al*., 2013). Therefore, for strategic, integrated conservation planning, we should consider that during most part of the year to lose a male may be as deleterious to a population as to lose a female.

Moreover, it is still unknown how vulnerable hoary foxes may be to climate changes, as termites’ swarming flights in the Cerrado occur between October and December, highly driven by rainfall and climate patterns (Prestes, 2012). Hence, changes in these factors could result in a mismatch between the hoary fox reproduction period and food availability, potentially reducing the offspring’s survival. Therefore, understanding the physiological mechanisms that drive reproductive seasonality will aid in the assessment of climate changes that could have an impact on this species.

Finally, our results indicate that longitudinal monitoring of faecal metabolites of oestrogen and progestagen can be used to differentiate reproductive from non-reproductive periods in female hoary foxes. This is the first study about the ovarian cycle on *Lycalopex vetulus* and also for the genus *Lycalopex*, providing valuable information about the reproductive biology of this South American group. We also highlight how basic physiological mechanisms can strongly contribute to conservation, from a better comprehension of species natural history to practical breeding planning in captivity. We suggest further studies regarding the reproductive physiology of hoary foxes. These should include the investigation in free-ranging animals of the oestrus cycle, adrenal function, male interaction, mating behaviour and physiological responses to environmental changes. These parameters will be essential for conservation planning and management of such peculiar and little-known Neotropical species.

## Funding

This work was supported in part by the Coordenação de Aperfeiçoamento de Pessoal de Nível Superior—Brasil (CAPES)—Finance Code 001 and by Fundação de Amparo à Pesquisa do Estado de São Paulo (FAPESP).
